# Wide range detector of plasma induced charging effect for advanced CMOS BEOL processes

**DOI:** 10.1186/s11671-021-03570-7

**Published:** 2021-07-03

**Authors:** Yi-Jie Chao, Kai-Wei Yang, Chi Su, Chrong-Jung Lin, Ya-Chin King

**Affiliations:** Institute of Electronics Engineering, National Tsing Hua University, Hsinchu, Taiwan

**Keywords:** Plasma induced damage, Advanced FinFET technology, Detection range

## Abstract

This work proposed a modified plasma induced charging (PID) detector to widen the detection range, for monitoring the possible plasma damage across a wafer during advanced CMOS BEOL processes. New antenna designs for plasma induced damage patterns with extended capacitors are investigated. By adapting the novel PID detectors, the maximum charging levels of the detectors have been enhanced.

## Introduction

In recent years, the evolution of semiconductor process technology continues to scale down the critical dimension in large-scale integrated circuits [1–3]. Advanced FinFET logic processes have become more complex for realizing more tightly packed transistors in multi-functional and more powerful Si chips. Reactive ion etching steps enhanced by plasma [4, 5] become inevitable in advanced nano-scale processes for achieving high aspect ratio structures which are essential for high packing density circuits [6]. For CMOS technology nodes beyond 45 nm, the transistor gates changed from the conventional poly-silicon gate with silicon dioxide to high-k metal gate stacks [7, 8]. This change makes the devices more susceptible to the plasma induced damage and might lead to unforeseen latent damages to the high-k dielectric layers. [9]. In state-of-art manufacturing processes of FinFETs, numerous RF plasma steps such as etching, deposition and cleaning processes are inevitable, which create higher frequencies of plasma induced charging events [10]. Both positive and negative charging on metal structures may occur. As these charges flow through the conductive paths made of pre-existing metal lines, via and contacts, the undesirable discharging through vulnerable parts of the circuits, particularly through the transistor gate dielectric may lead to significant reliability concerns. For instance, in the dry etching step, scattering impinging ions and sputtered materials at the reaction surface cause more defects in the bulk fins [11, 12]. To avoid the plasma charging event leading to irreversible damage to circuits, design rules that limit the size of metal structures are given. Another example of alleviating PID includes using protective diodes, which could shunt the plasma charging current away from sensitive circuits [13]. The introduction of In-Situ Steam Generation (ISSG) gate oxide reported improving its tolerance for plasma damage [14]. Furthermore, trimming the chamber and modifying PECVD-Ti deposition process were also found to alleviated plasma induced damage [15]. Most of these methods however result in undesirable limitations on circuit design flexibility or processing tradeoffs.

Conventionally, on-wafer test patterns have been used to monitor the plasma induced damage (PID) levels [16]. The most common and widely used parameter for monitoring on-wafer PID is the time-to-breakdown (TDDB) characteristics of the transistor gates with large antenna structures. The latent damage on gate dielectrics can be revealed by measuring the degradation of the gate dielectric layer under voltage or current stress tests. Hence, these patterns are not able to provide real-time feedback on the plasma processes [17]. In our previous works, an on-wafer plasma induced charging effect detector is demonstrated in advanced FinFET technologies. The PID detector uses capacitive coupling structure to induce a response on the floating gate [18–20]. Therefore, there is no damage to the gate dielectric layer as it does in a conventional PID detector. On these new detectors, one measures the shifting I–V curves to find out both the intensity, duration as well as polarity of charges on the antenna gate. It is found that these detectors may subject to saturation effect as the plasma intensity at certain recording sites exceeds critical levels. To extend the dynamic range of the PID detector, new antenna gate designs have been investigated in this work, where widening of the sensing ranges is successfully demonstrated.

## Methods

**Fig. 1:**
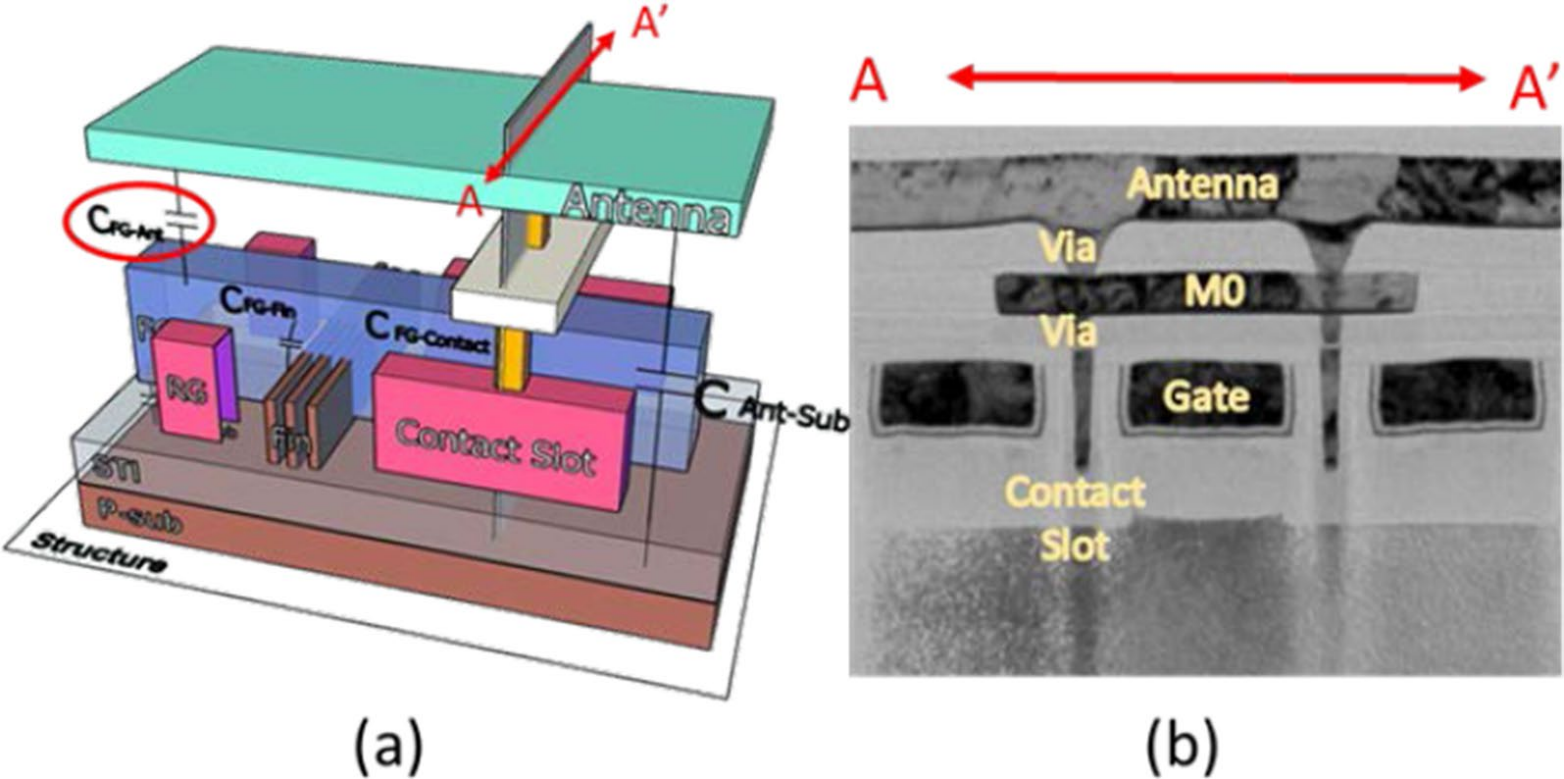
**a** The 3D illustrations of previously reported in-situ PID detector and the antenna capacitor is highlighting in this structure. **b** The TEM photo of PID detector, where the gate length of this detector is 140 nm

The 3D schematic of plasma induced damage (PID) detector with a parasitic capacitor connected to the antenna node is shown in Fig. 1a. Differing from PID monitoring structure, this detector utilizes a long contact slot to couple the antenna voltage on the floating gate. The cross-sectional TEM photograph is shown in Fig. 1b. As shown in the figure, contact slots which collect charges are capacitively coupled to floating gate.

**Fig. 2:**
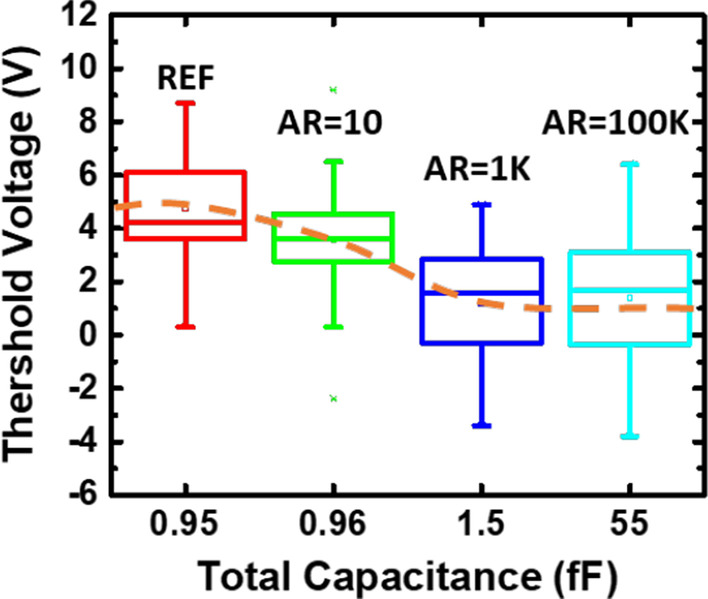
Threshold voltage range of samples with different size of antenna, and the corresponding total capacitance on the antenna

Figure 2 compares the recorded threshold voltage distributions from these detectors across a 12-inches wafer. The negative threshold voltage shift indicates that negative charges were collected on the antenna, drawing positive charges into floating gate, resulting in negative threshold voltage shifts. It is found that as the antenna area increases, the rising total capacitance leads to lowering of the overall antenna voltages, hence, smaller the shift in *V*_t_.

**Fig. 3:**
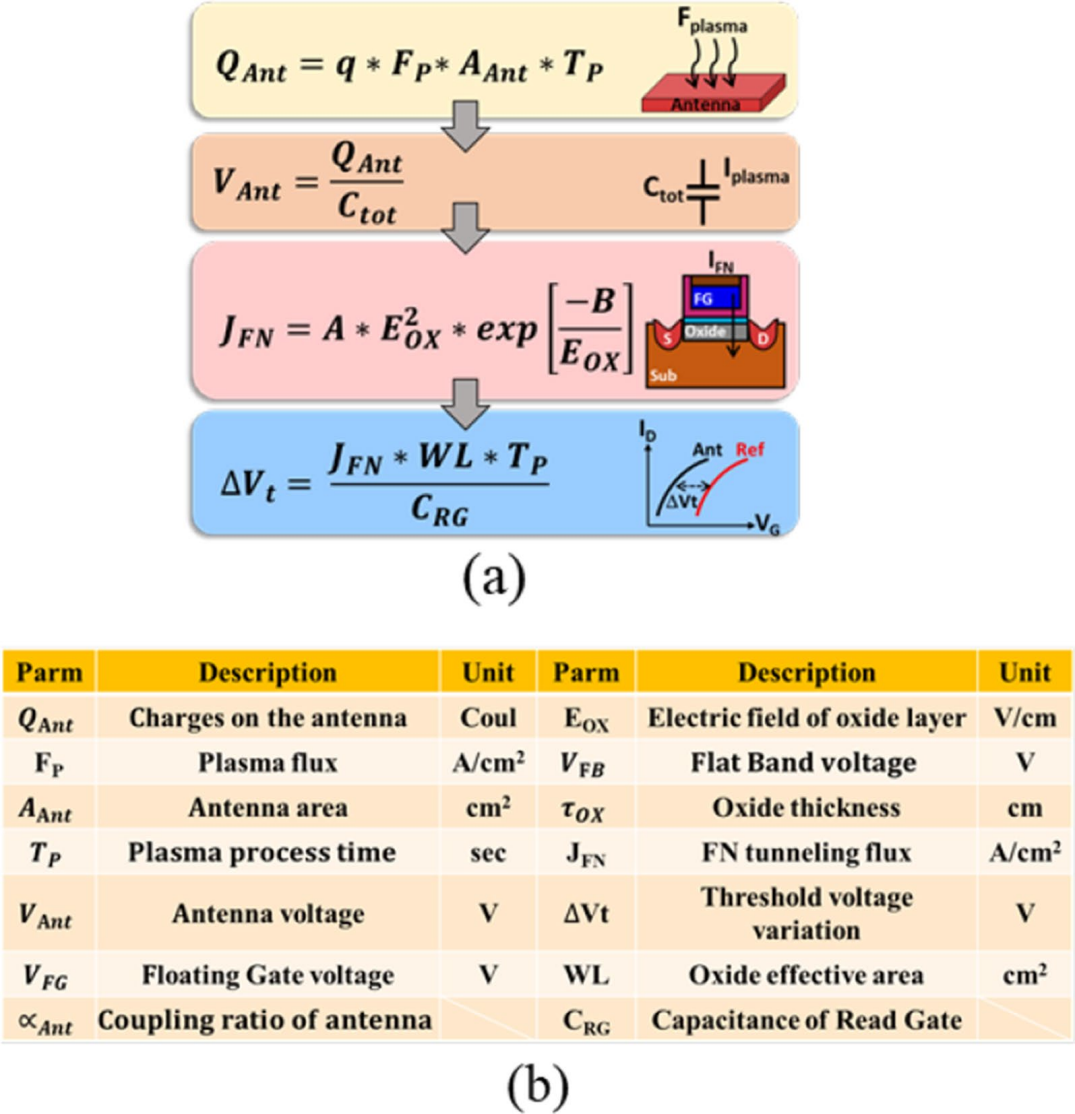
**a** The flow chart from plasma charges (*Q*_Ant_) collected on the antenna to shift *V*_t_. Based on the FN tunneling model, Δ*V*_t_ can be calculated. **b** The list of parameters with its definitions

Here, in Fig. 3a, the flow chart explaining the basic operation principles of the PID detector is outlined. As the plasma charge (*Q*_Ant_) are collected on the antenna, the potential of the antenna gate, *V*_Ant_, varies. *V*_Ant_ is then coupled to the floating gate (FG), promoting the tunneling of electrons either into or out of FG. After plasma processes, *V*_t_ of these detectors may become more negative or more positive based on the polarity of *Q*_Ant_. *V*_t_ can be calculated by the FN tunneling current model with the parameter listed in Fig. 3b.

**Fig. 4:**
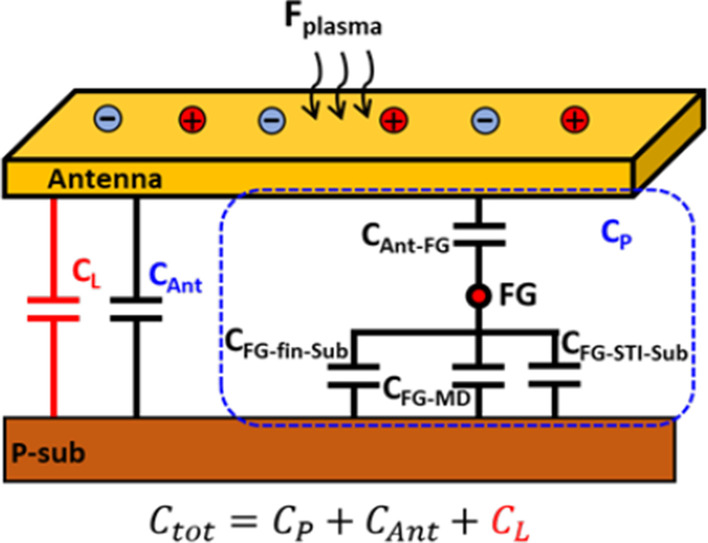
Composition of capacitance on the antenna structure with the additional loading capacitor, which is designed to modify the sensitivity of the PID detectors. Where *C*_P_ is the overall parasitic capacitance on the floating gate

**Fig. 5:**
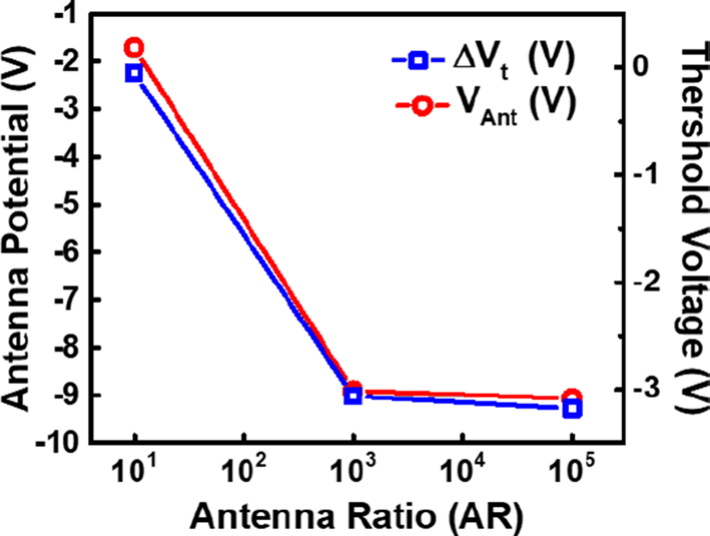
Both the threshold voltage level as well as projected antenna voltage level saturates on patterns with increased antenna area

Figure 4 illustrates all the possible capacitance on the FG-based PID detector. From Fig. 5, it is found that as the antenna area increases, Δ*V*_t_ tends to saturate. As *V*_Ant_ reaches the maximum levels, *Q*_Ant_ starts to leak out when the voltage level is too high. To avoid the plasma flux level exceeding the detector limitation, the antenna capacitance is deliberately increased by adding loading capacitors which could reduce the proportion of antenna capacitance in the total capacitance.

**Fig. 6:**
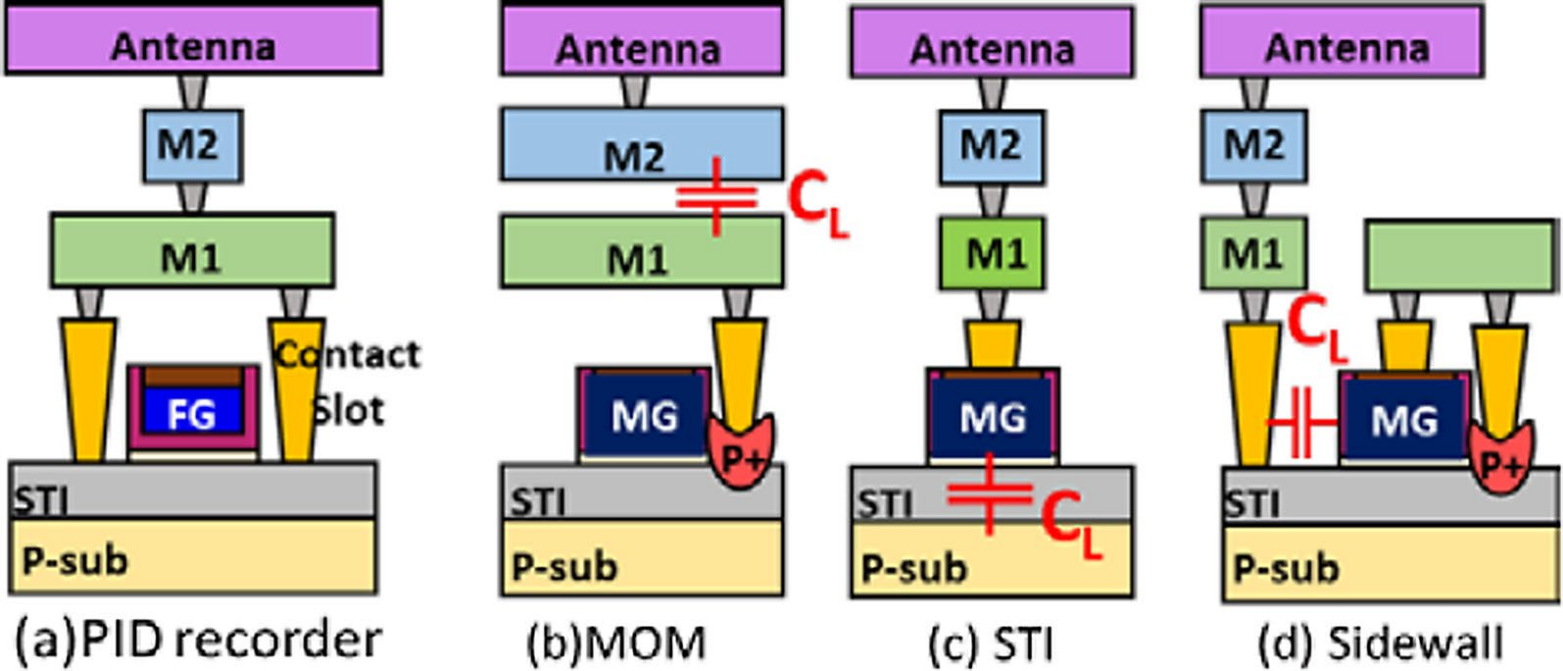
Cross sectional illustration of **a** the in-situ PID detector, and those with a loading additional capacitor realized by **b** MOM, **c** STI, and **d** sidewall, respectively

Figure 6a shows the 2D structure of the previously reported in-situ PID detector, and three structures of realizing additional capacitors are presented. They are MOM capacitors, which use the larger overlap area of metal layers to increase the total capacitance in Fig. 6b, STI capacitors, which increase the capacitance by adjusting the length of the metal gate in Fig. 6c, and sidewall capacitors, which use the overlap area of metal gate and contact to form additional capacitor Fig. 6d.

## Experimental results and discussion

**Fig. 7:**
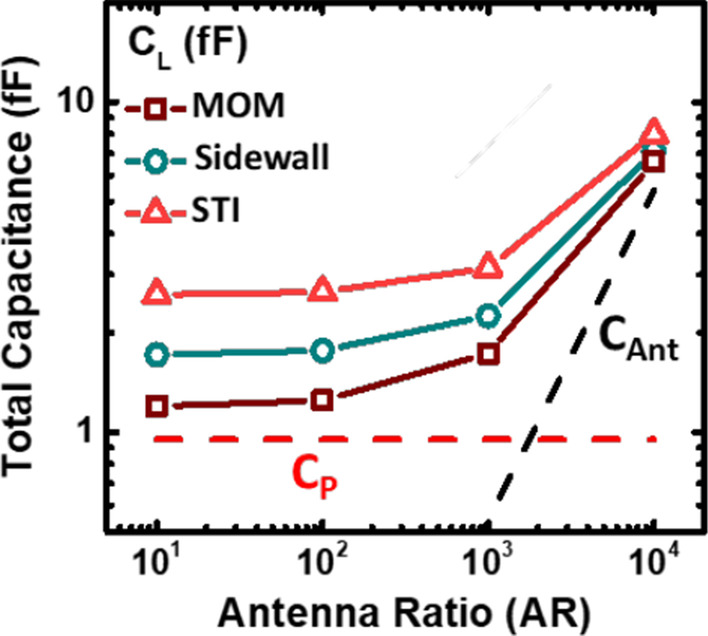
Comparison of total capacitance versus antenna ratios as the three types of loading capacitors are added

Figure 7 compares the total capacitance versus antenna ratios when different types of loading capacitors are added. The total capacitance is dominated by the antenna capacitance when the antenna ratio is greater than 1 K.

**Fig. 8:**
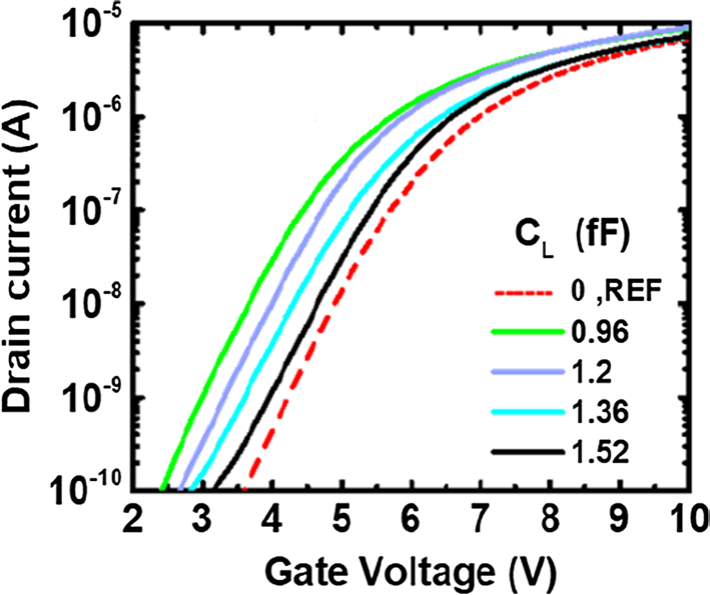
I–V curve of different sizes of STI capacitor with AR = 10. When the external capacitance is larger, the I–V curve is closer to that of the reference cells

When the added loading capacitance become comparable to the antenna capacitance, total capacitance could then be affected by the loading capacitors. Since the maximum amount of charges collected by the antenna is fixed; by increasing total capacitance, Δ*V* is expected decreased, according to Δ*Q* = *C*Δ*V*. Thus, the sensitivity of the detection range could be suppressed, preventing saturation effect when plasma charging level exceed its original limits. Drain current characteristics for devices of AR = 10 with different STI capacitors are compared in Fig. 8. When a larger loading capacitor is added, the percentage of antenna capacitance in total capacitance is reduced. Under the same plasma charging flux, the total plasma charges after a period is proportional to the antenna area. Hence, when overall capacitance increases, *V*_Ant_ is expected to be lowered, allowing for the detection of high plasma flux levels. As shown in Fig. 8, smaller shifts are found on the I–V curves for the samples with additional loading capacitors.

**Fig. 9:**
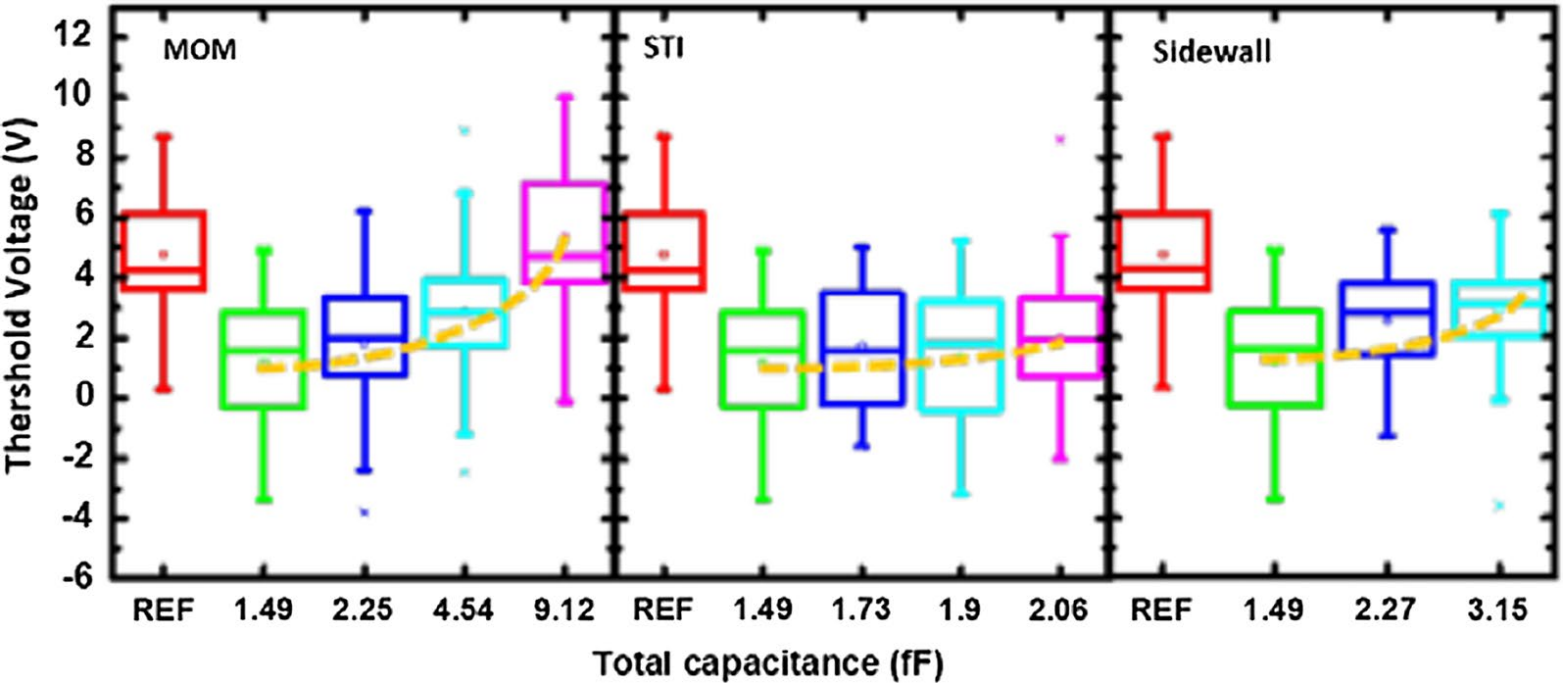
Threshold voltages measured on samples with different sizes of MOM, STI and Sidewall capacitors are compared. All devices have the same AR of 1 K

**Fig. 10:**
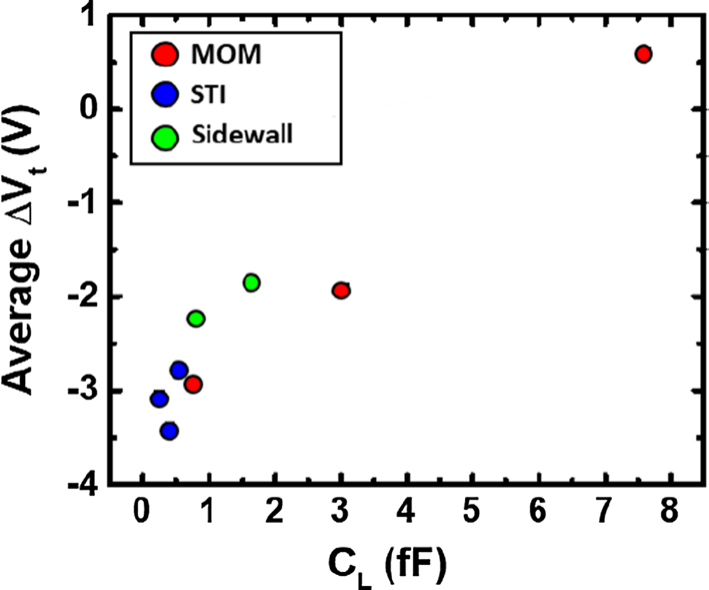
Comparison of the average Δ*V*_t_ versus *C*_L_ implemented by the three types of capacitor structures, where Δ*V*_t_ is defined as the *V*_t_ difference between a detector cell and the reference cell. Data suggest that reduced sensitivity can be obtained as *C*_L_ increased

Box charts of the threshold voltages measured from samples with AR = 1 K and different sizes of MOM, STI, and Sidewall capacitors are compared in Fig. 9 When the loading capacitance is increased, less threshold voltage shift is observed on average. In the experimental design, *C*_L_ by STI structure is too small to show impact of the charging level. Comparison in Fig. 10 suggested that three ways of adding loading capacitors can also effectively reduce the average response to plasma charging. The additional loading capacitor can successfully expand the detection range of the PID detector, while the sensitivity of the detectors is reduced. For achieving wide-range detection of plasma charging level, a series of PID detectors with different level of *C*_L_ can be designed in a 1-D array for detecting plasma charging levels on both the high and low end.

## Conclusions

This study investigates a new antenna gate design to extend the sensing range of plasma induced charging levels on the PID monitoring detectors. By adding a loading capacitor, high antenna gate voltage subject to charge leak can be prevented, allowing for a higher level of charging level to be registered on the PID detectors. This novel design effectively widens the detection range of plasma charging levels in advanced CMOS BEOL processes.

## Data Availability

Not Applicable.

## Abbreviations

PID: Plasma induced damage
*V*_t_: Threshold voltage
*Q*_Ant_: Charges on the antenna
*V*_Ant_: Antenna voltage
MOM: Metal-oxide-metal
AR: Antenna ratio
*C*_L_: Loading capacitance
*C*_P_: Parasitic capacitance
*C*_Ant_: Antenna capacitance
